# Enriched endoplasmic reticulum-mitochondria interactions result in mitochondrial dysfunction and apoptosis in oocytes from obese mice

**DOI:** 10.1186/s40104-017-0195-z

**Published:** 2017-08-01

**Authors:** Lihong Zhao, Tengfei Lu, Lei Gao, Xiangwei Fu, Shien Zhu, Yunpeng Hou

**Affiliations:** 1 State Key Laboratory of Agrobiotechnology, College of Biological Sciences, China Agricultural University, Yuanmingyuan west Rd 2, Beijing, 100193 China; 2 Key Laboratory of Animal Genetics, Breeding and Reproduction, College of Animal Science and Technology, China Agricultural University, Beijing, China

**Keywords:** Apoptosis, IP3R1, MAM, Mitochondrial Ca^2+^, Obesity, Oocyte, PACS-2

## Abstract

**Background:**

Maternal obesity alters oocytes and subsequent fetal metabolism. An increasing number of studies have shown that the endoplasmic reticulums (ER) or mitochondria have important effects on oocyte quality, but there has been no study of the effect of mitochondria-associated ER membranes (MAMs) on oocyte quality. The present study was designed to assess whether the level of MAM and MAM-related proteins were different in oocytes from obese and control mice.

**Results:**

First, oocytes from mice with high-fat-diet (HFD)-induced obesity had higher levels (either greater numbers or a higher proportion for the same numbers) of MAM than oocytes from control mice. The abundance of MAM-related proteins in oocytes from obese mice was significantly greater at both the messenger RNA and protein levels, including inositol 1,4,5-trisphosphate receptor, type 1 (IP3R1), inositol 1,4,5-trisphosphate receptor, type 2 (IP3R2) and phosphofurin acidic cluster sorting protein 2 (PACS-2). Further, there was an increase in mitochondrial Ca^2+^ ([Ca^2+^]_m_) which was associated with increased apoptosis and compromised cytoplasmic maturation in oocytes from obese mice. Down-regulation of MAM-related protein IP3R1 in oocytes from obese mice decreased [Ca^2+^]_m_ and apoptosis and improved cytoplasmic maturation but did not reduce the overall MAM level. However, down-regulating MAM-related protein PACS-2 in oocytes from obese mice did reduce the level of MAM and [Ca^2+^]_m_, which decreased the rate of apoptosis and improved cytoplasmic maturation of oocytes from obese mice.

**Conclusions:**

It is possible that enriched MAM could increase [Ca^2+^]_m_, and this increase has been found to be associated with increased apoptosis and compromised cytoplasmic maturation in oocytes from obese mice. This finding suggests a novel therapeutic target for obesity-induced oocyte defects.

**Electronic supplementary material:**

The online version of this article (doi:10.1186/s40104-017-0195-z) contains supplementary material, which is available to authorized users.

## Background

Obesity in females of reproductive age not only increases their risks of developing insulin resistance, diabetes, and other diseases, but is also linked to poor reproductive outcome [1–3]. Oocyte quality plays a key role in connecting maternal nutrition with reproductive outcome, and is dramatically impacted by maternal obesity [4, 5]. A previous report demonstrated that obese women have a lower success rate in both natural and assisted reproduction. Moreover, the higher pregnancy failure rate in obese women returns to normal if they use donor oocytes to replace autologous oocytes [6]. Although the molecular mechanisms by which oocytes are impaired by maternal obesity are not completely understood, dysfunctions of the endoplasmic reticulum (ER) or mitochondria have been strongly implicated [7–9].

The ER is the key calcium (Ca^2+^) storage and release system [10]. Obesity changes the ER membrane lipid composition and damages its ability to retain Ca^2+^, which alters the cytoplasmic homeostasis [11]. Mitochondria are the primary effectors of Ca^2+^ absorption and intrinsic apoptosis [12]. Prolonged Ca^2+^ release from the ER leads to altered mitochondrial membrane potential and the induction of intrinsic apoptotic pathways [13, 14]. Mitochondrial defects are thought to be the direct reason for the oocyte developmental deficits in obese females [15].

The ER and mitochondria interact both physiologically and functionally via mitochondria-associated ER membranes (MAM). MAM is involved in a number of physiological processes including Ca^2+^ transfer and cellular apoptosis [16–18]. MAM contains several proteins such as inositol 1,4,5-trisphosphate receptor, type 1 (IP3R1; a major Ca^2+^ release channel in the ER), inositol 1,4,5-trisphosphate receptor, type 2 (IP3R2), mitofusin-2 (Mfn2), phosphofurin acidic cluster sorting protein 2 (PACS-2; controls the apposition of mitochondria with the ER), sigma-1 receptor (Sig-1R), and calnexin. A recent study demonstrated there to be significantly more MAMs in the livers of obese mice compared with control mice [19].

To date, the association between MAM and altered oocyte quality in obese mice has not been investigated. The current study investigated whether the content of MAM and MAM-related proteins were different in oocytes from obese and control mice.

## Methods

All chemicals were purchased from Sigma Chemical Co (St Louis, MO, USA) unless otherwise stated.

### Obese mice and oocyte harvesting

Female CD-1 mice (3-week-old) were purchased from the Beijing Vital River Experimental Animals Centre (Beijing, China) and housed under 12 h: 12 h light: dark cycles at a temperature of 23 ± 2 °C for all experiments. The mice were randomly divided into two groups (five per cage): one group was fed a control diet (CD) and the other was fed a high fat diet (HFD) for 12 week (the formulae of the two diets are shown in Additional file 1: Table S1); water was provided ad libitum. After obesity had been established (Additional file 2: Fig. S1), mice from the two groups were fasted overnight and weighed. Before all experiments, the mice were treated with 8 IU of equine chorionic gonadotropin (eCG) for 46–48 h and then sacrificed by cervical dislocation. Germinal vesicle (GV) oocytes were collected for subsequent experiments. All procedures were performed under the Institutional Animal Care and Use Committee of China Agricultural University.

### Transmission electron microscopy (TEM)

Oocytes were collected and fixed with a fixative buffer (1% paraformaldehyde, 2.5% glutaraldehyde in 0.1 mol/L PBS, pH 7.4) for 3 h at 4 °C. They were then washed in 0.1 mol/L PBS, post-fixed with 1% osmium tetroxide (Ted Pella, Redding, CA, U.S.) and 1.5% potassium ferricyanide. Samples were dehydrated with a graded alcohol series, placed in 100% acetone, and embedded in 100% 812 resin. Ultrathin slices (70 nm) generated by an ultramicrotome were stained with 2% uranyl acetate and viewed under a TEM (H-7650, Hitachi, Tokyo, Japan). The main structures in a larger area of oocytes from the CD and HFD groups were depicted using Imaris software (Bitplane, Concord, NH, U.S.).

### Quantitative real-time PCR (qRT-PCR) and semi-quantitative reverse transcription PCR (RT-PCR)

Total RNA was extracted from collected oocytes using an RNeasy micro-RNA isolation kit (Qiagen, Valencia, CA, U.S.) following the manufacturer’s instructions. The RNA concentrations were measured using a Nanodrop ND-1000 Spectrophotometer (Biolab, Scoresby, Victoria, Australia). Reverse transcription was conducted to generate cDNA libraries using a QuantiTect Reverse Transcription Kit (Qiagen) according to the manufacturer’s instructions. QRT-PCR and RT-PCR were performed using an ABI 7500 real-time PCR instrument or a Veriti 96-well Thermal Cycler (Applied Biosystems, Foster City, CA, U.S.). The sequences of all primers used are listed in Additional file 3: Table S2. The results were analyzed using the 2^−ΔΔCt^ method.

### Western blotting

Oocytes were washed in PBS three times, collected, and homogenized in sodium dodecyl sulfate buffer. They were then boiled for 5 min and immediately cooled on ice. The proteins were separated using SDS-PAGE with a 5% stacking gel and a 6% (for proteins greater than 90 kDa) or 10% (for proteins less than 90 kDa) separating gel and transferred to nitrocellulose membranes. After blocking with Tris-buffered saline with Tween-20 (TBST) containing 5% BSA, the membranes were incubated with the following primary antibodies: anti-IP3R2, anti-IP3R1 (1:1,000; Thermo Fisher Scientific, Rockford, IL, U.S.), anti-PACS2 (1:500; EMD Millipore, Temecula, CA, USA), anti-actin (1:200; Santa Cruz Biotechnology, Santa Cruz, CA, U.S.), anti-p-MAPK3/1, anti-MAPK3/1 (1:2,000), anti-cytochrome C (1:5,000), anti-caspase 3 (1:1,000; Cell Signaling Technology, Beverly, MA, U.S.), and anti-GAPDH (1:500; CW Biotech, Beijing, China) in TBST overnight at 4 °C. The membranes were incubated with horseradish peroxidase (HRP)-conjugated secondary antibodies (1:5000, CW Biotech) for 1 h at 25 °C. Subsequently, the blots were detected using a chemiluminescence kit (Thermo Fisher Scientific). All results were quantified using ImageJ software (National Institutes of Health, Bethesda, MD, U.S.).

### Measuring mitochondrial Ca^2+^ ([Ca^2+^]_m_)

[Ca^2+^]_m_ levels were assessed using Rhod-2AM (Invitrogen/Molecular Probes, Carlsbad, CA, U.S.) according to a previous study [20]. Briefly, zona pellucid was enzymatically removed. The oocytes were then processed in maturation medium with 3 μmol/L Rhod-2AM for 30 min, rinsed three times, and incubated without Rhod-2AM at 37 °C and 5% CO_2_ for 30 min. Subsequently, they were analyzed using a confocal laser scanning microscope (Nikon A1R, Tokyo, Japan) and quantitatively processed using NIS-Elements AR (Nikon Instruments, Tokyo, Japan).

### Measuring intracellular ROS and GSH levels

Intracellular ROS and GSH levels were measured as described previously [21]. Oocytes were incubated in M_2_ supplemented with 1 mmol/L 2′, 7′-dichlorodihydrofluorescein diacetate (H_2_DCFDA) for measuring ROS or 10 μmol/L 4-chloromethyl-6,8-difluoro-7-hydroxycoumarin (CellTracker Blue) for measuring GSH for 30 min at 37 °C and washed three times. The fluorescence was examined under an epifluorescence microscope with a filter at 460-nm excitation for ROS and 370-nm excitation for GSH (DP72, Olympus, Tokyo, Japan). All data were analyzed using ImageJ software.

### siRNA microinjection

siRNAs were microinjected into oocytes as described previously [20]. To knockdown *Itpr1* or *Pacs-2*, 10 μmol/L of each siRNA smart pool (a mixture of four siRNAs) or NC siRNA (GE Healthcare Life Sciences, South Logan, UT, U.S.) was used and the microinjection was completed within 30 min. All oocytes were arrested at the GV stage and cultured in maturation medium supplemented with 4 mol/L hypoxanthine (HX) to deplete the endogenous targeted mRNAs and proteins. The sequences of *Itpr1* siRNAs were as follows: siRNA-1: 5′-GAAAUGGAGUGAUAACAAA-3′; siRNA-2: 5′-GGACGAGGCUGGAAAUGAA-3′; siRNA-3: 5′-GUACAUGAGUUCUUCUAUA-3′; siRNA-4: 5′-GAGAUGAACUGGCAGAAGA-3′. The sequences of *Pacs-2* siRNAs were as follows: siRNA-1: 5′-GCUCAGGCCUUACUUCGAA-3′; siRNA-2: 5′-CCAACAGCCUAGACAAUGA-3′; siRNA-3: 5′-GGACGAUCCUGGGCUACAA-3′; siRNA-4: 5′-UCUCUCGGAUACAGCGAUA-3′. The sequences of NC siRNAs were as follows: siRNA-1: 5′-UAGCGACUAAACACAUCAA-3′; siRNA-2: 5′-UAAGGCUAUGAAGAGAUAC-3′; siRNA-3: 5′-AUGUAUUGGCCUGUAUUAG-3′; siRNA-4: 5′-AUGAACGUGAAUUGCUCAA-3′.

### Statistical analyses

Each experiment was repeated at least three times. A representative image of each experiment is shown. All data are presented as means ± SEM and were analyzed using *t*-tests with SigmaPlot (Systat Software, San Jose, CA, U.S.U.S.). Differences were deemed statistically significant as follows: * *P* < 0.05, ** *P* < 0.01.

## Results

### HFD-induced obesity increases the amount of MAM in mouse oocytes

**Fig. 1:**
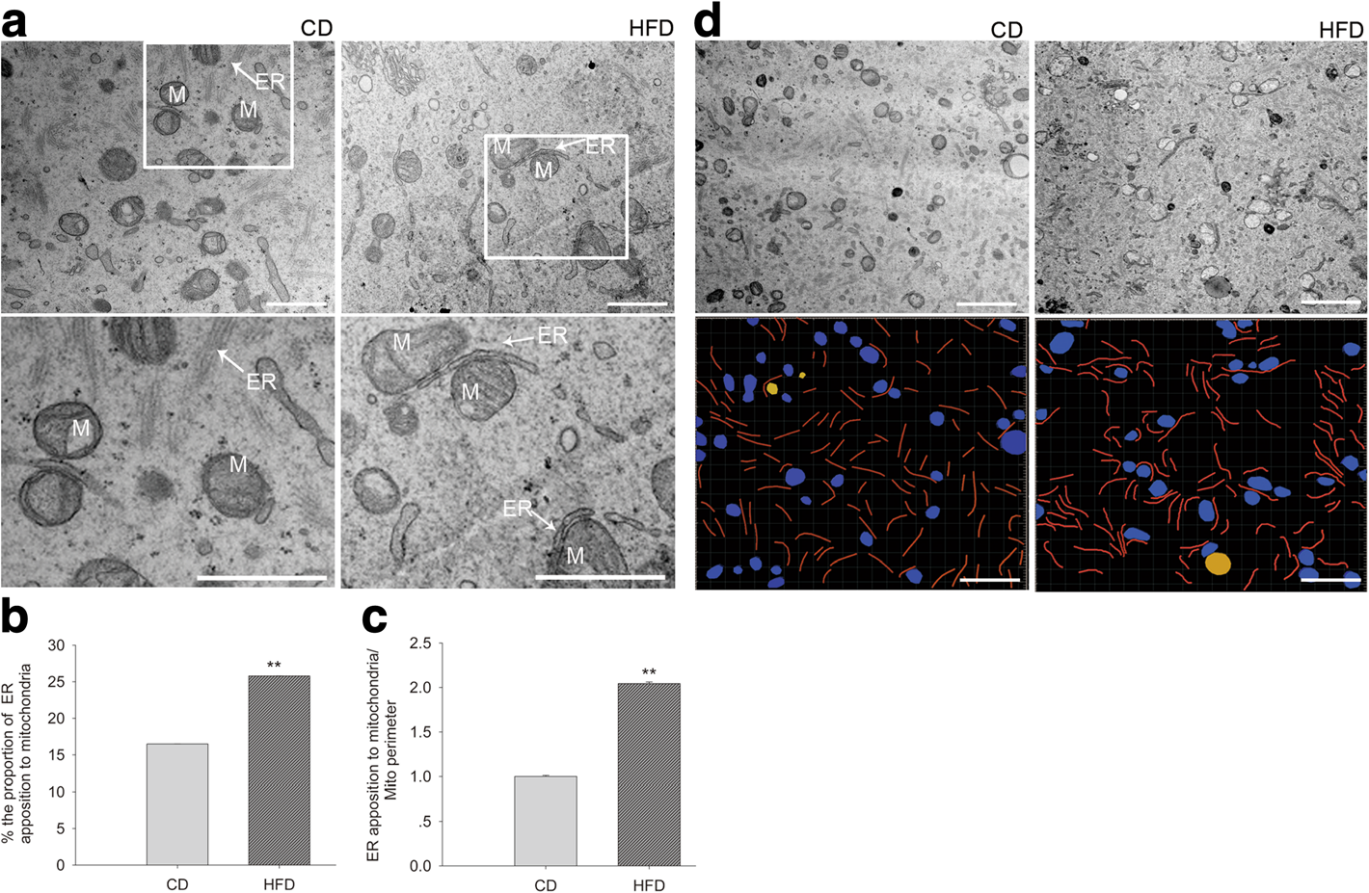
HFD induced obesity leads to increased content of MAM in mouse oocytes. **a** Characteristic TEM images of oocytes from control or obese mice. Magnification, 30,000×; scale bars, 500 nm. The lower panels showed a higher magnification of the selected areas in the top panels. ER, endoplasmic reticulum; M, mitochondria; N, nucleus. **b**, **c** The length of the ER tethered to the mitochondria was normalized to the ER length (**b**) or mitochondrial circumference (**c**). *N* = 30 (five sections per mice, six mice per group) for each group across three replicates. **d** The subcellular structures of a larger area of oocytes from both groups are depicted. Magnification, 15,000×; scale bars, 1 μm. Graphs show means ± SEM. * *P* < 0.05, ** *P* < 0.01, CD vs. HFD; *t*-test

The ER and mitochondria were in closer proximity to each other in oocytes from obese mice than in those from control mice (Fig. 1a). The proportion of the physical apposition relative to the total ER length (Fig. 1b) or mitochondrial circumference (Fig. 1c) was significantly greater in oocytes from obese mice. For a better view of the alterations to the MAMs in oocytes from obese mice, a simple corresponding subcellular structural diagram of the ER, mitochondria, and lipid droplets was generated (Fig. 1d) within a larger field of vision compared with that shown in Fig. 1a.

### Oocytes from obese mice express more MAM-related genes and proteins

**Fig. 2:**
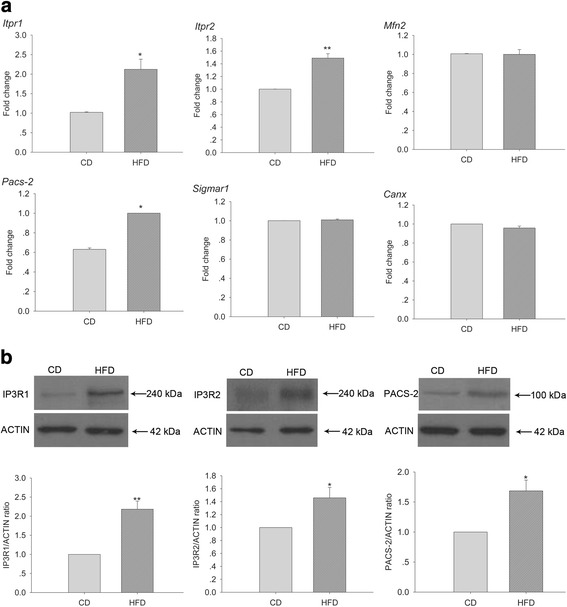
Oocytes from obese mice exhibit altered MAM-related gene and protein expression. **a** QRT-PCR showing *Itpr1*, *Itpr2*, *Pacs-2*, *Mfn2*, *Sigmar1*, and *Canx* mRNA levels. *N* = 240 for each group across three replicates. **b** Western blotting showing IP3R1, IP3R2, and PACS-2 protein levels. The lower histograms show quantification of the upper panels. *N* = 450 for each group across three replicates. Graphs show means ± SEM. * *P* < 0.05, ** *P* < 0.01, CD vs. HFD; *t*-test

To assess any changes in MAM-related proteins in oocytes from obese mice, the mRNA and protein levels of these proteins in oocytes were measured. Notably, oocytes from obese mice exhibited significantly higher IP3R1, IP3R2, and PACS-2 expression at both the mRNA (*Itpr1*, *Itpr2*, and *Pacs-2*) (Fig. 2a) and protein levels (Fig. 2b).

### Oocytes from obese mice exhibit higher [Ca^2+^]_m_ levels and increased apoptosis

**Fig. 3:**
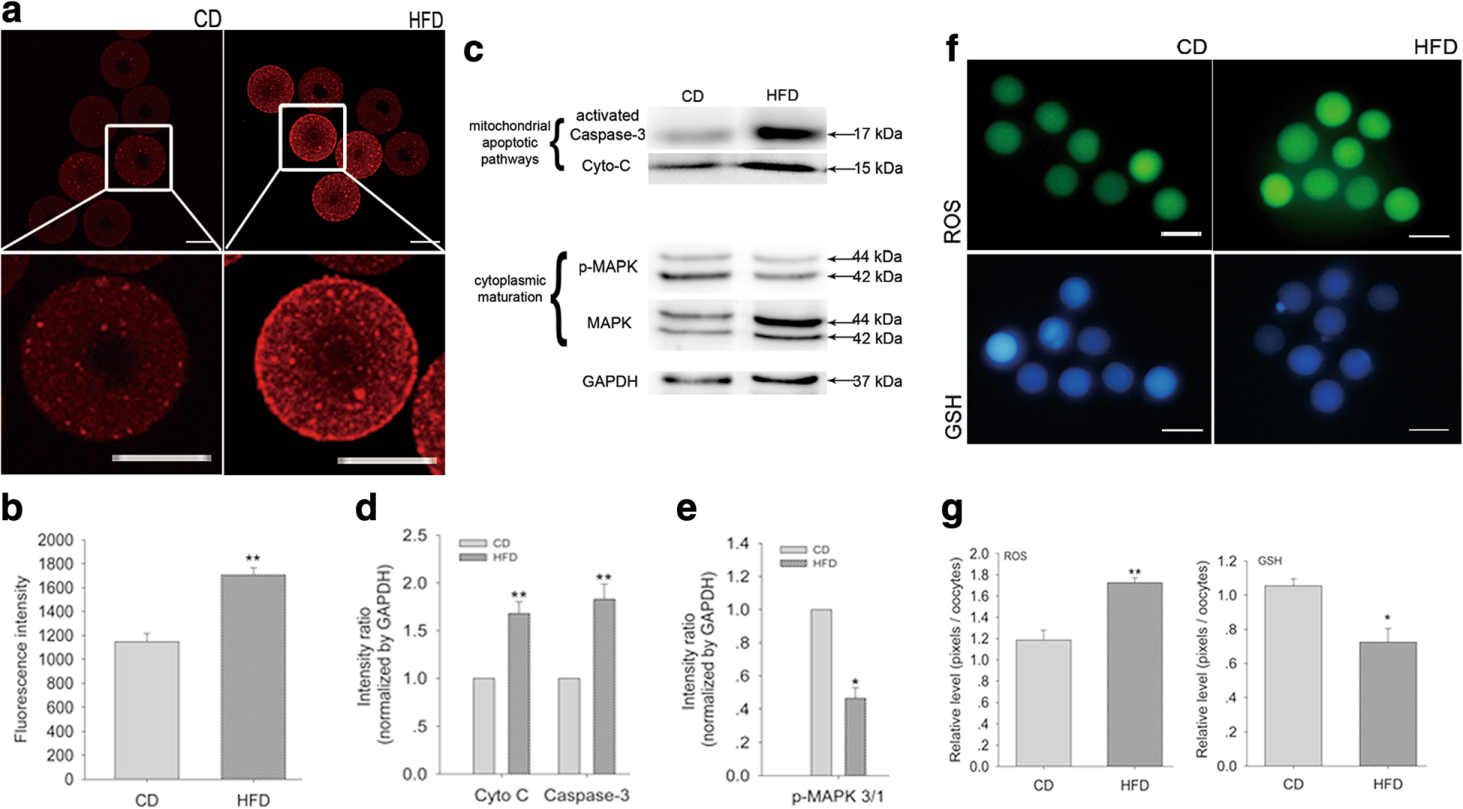
Oocytes from obese mice exhibit higher [Ca^2+^]_m_ and increased apoptosis. **a** [Ca^2+^]_m_ levels in oocytes from control and obese mice. Scale bars, 50 μm. **b** Quantitative analysis of [Ca^2+^]_m_. *N* = 30 per group across three replicates. **c** Western blotting for activated caspase-3, cytochrome C, P-MAPK-3/1, and MAPK-3/1. **d** Statistical analysis of activated caspase-3 and cytochrome C levels. **e** Statistical analysis of P-MAPK-3/1 and MAPK-3/1 levels. *N* = 360 per group across three replicates. **f** ROS (green) and GSH (blue) levels in control and obese mice. Scale bars, 100 μm. **g** Quantitative analysis of ROS and GSH levels. *N* = 30 per group across three replicates. Graphs show means ± SEM. * *P* < 0.05, ** *P* < 0.01, CD vs. HFD; *t*-test

[Ca^2+^]_m_ levels were increased in oocytes from obese mice at baseline (Fig. 3a). Quantitative analysis revealed that the mean [Ca^2+^]_m_ intensity was significantly higher in oocytes from obese mice compared with those from control mice (Fig. 3b).

Mitochondrial physiology is very sensitive to alterations in [Ca^2+^]_m_, and abnormal increases in Ca^2+^ could activate the signaling pathways related to oocyte apoptosis. Indeed, the expression of cytochrome C and activated caspase-3 was greatly increased in oocytes from obese mice (Fig. 3c, d). In addition, the phosphorylation of extracellular-regulated protein kinase 3/1 (P-MAPK 3/1) (Fig. 3c, e) and GSH levels (Fig. 3f, g) were significantly decreased and ROS levels were significantly increased (Fig. 3f, g) in oocytes from obese mice.

### Knocking down IP3R1 prevents the metabolic decline in oocytes from obese mice

**Fig. 4:**
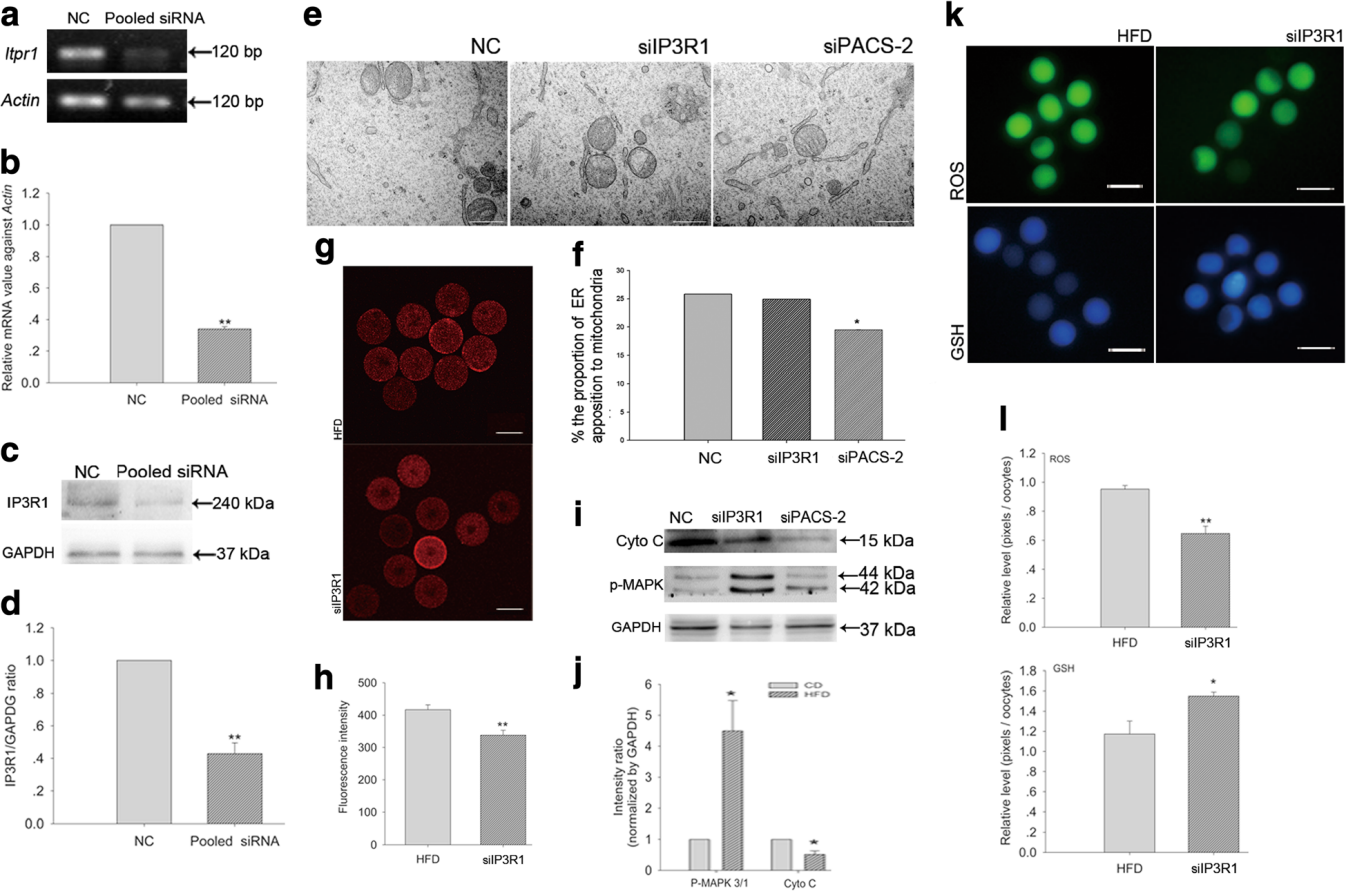
Knocking down IP3R1 prevents the metabolic decline in oocytes from obese mice. **a** The mRNA levels of *Itpr1* after knocking down IP3R1. **b** The relative mRNA levels of *Itpr1*. *N* = 90 per group across three replicates. **c** Representative immunoblots showing IP3R1 protein levels after IP3R1 knockdown. **d** The relative protein levels of IP3R1. *N* = 300 per group across three replicates. **e** Characteristic TEM images of oocytes from NC, siIP3R1 and siPACS-2 treatment. Magnification, 60,000×; scale bars, 500 nm. **f** The length of the ER tethered to the mitochondria was normalized to the ER length. *N* = 30 (five sections per mice, six mice per group) for each group across three replicates. **g** [Ca^2+^]_m_ levels after IP3R1 knockdown; scale bars, 50 μm. **h** Quantitative analysis of [Ca^2+^]_m_. *N* = 30 per group across three replicates. **i** Representative immunoblots of increased P-MAPK 3/1 and decreased cytochrome C levels after IP3R1 or PACS-2 knockdown (left column, NC siRNAs; middle column, *Itpr1* pooled siRNAs; right column, *Pacs-2* pooled siRNAs). **j** Quantification of the immunoblots. *N* = 300 per group across three replicates. **k** ROS and GSH levels after IP3R1 knockdown; scale bars, 100 μm. **l** Statistical analysis of ROS and GSH levels after IP3R1 knockdown. *N* = 30 per group across three replicates. Graphs show means ± SEM. * *P* < 0.05, ** *P* < 0.01, CD vs. HFD; *t*-test

siRNA treatment decreased both the mRNA (Fig. 4a, b) and protein (Fig. 4c, d) expression of IP3R1. The level of MAM was not influenced by IP3R1 knockdown (Fig. 4e, f), but the [Ca^2+^]_m_ was reduced (Fig. 4g, h), the levels of cytochrome C was reduced and the levels of P-MAPK 3/1 increased (Fig. 4i, j). This was accompanied by decreased ROS levels and increased GSH levels (Fig. 4k, l).

### Knocking down PACS-2 alleviates the metabolic decline in oocytes from obese mice

**Fig. 5:**
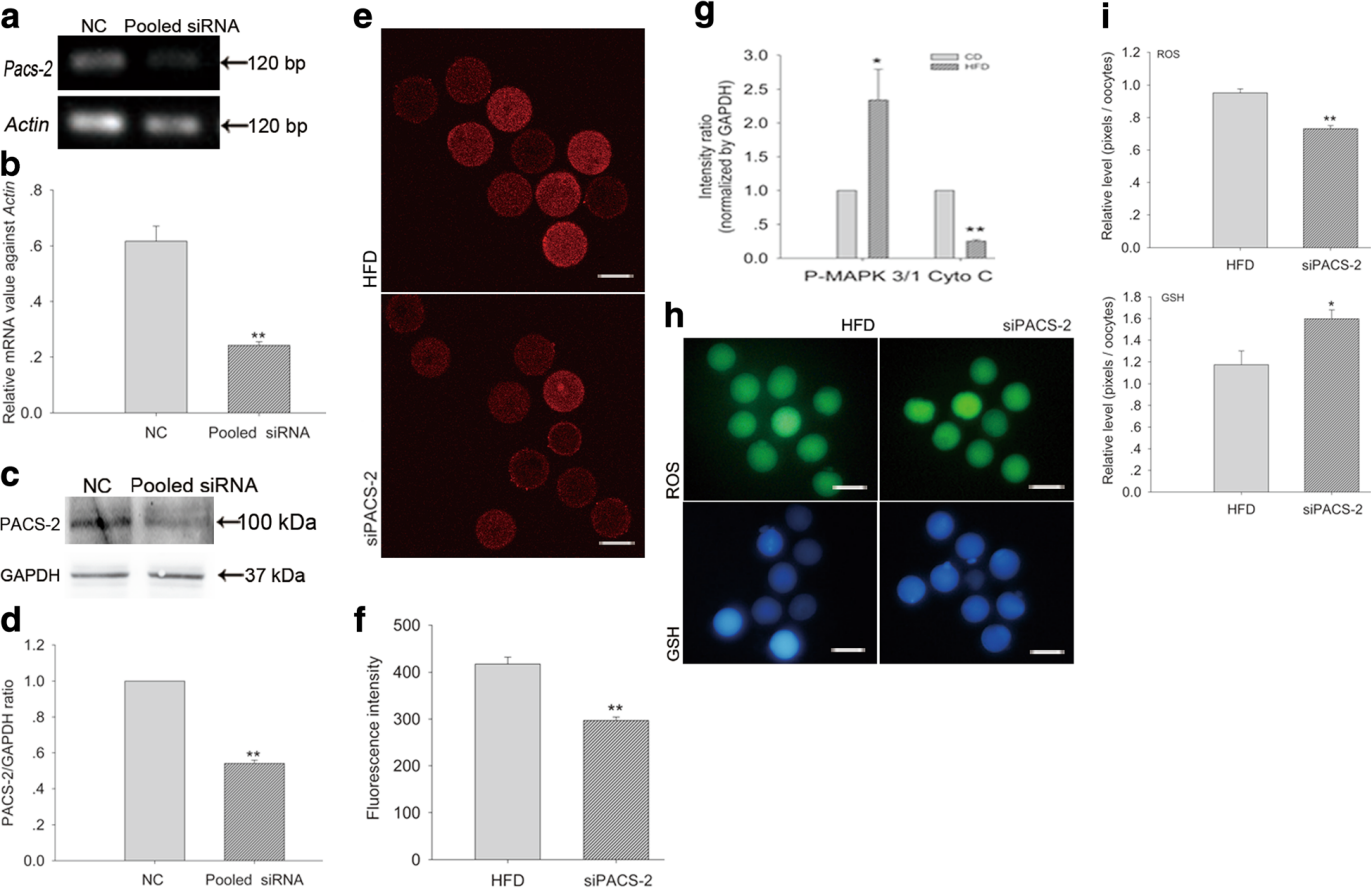
Knocking down PACS-2 alleviates the metabolic decline in oocytes from obese mice. **a** The mRNA levels of *Pacs-2* after PACS-2 knockdown. **b** The relative mRNA levels of *Pacs-2*. *N* = 90 per group across three replicates. **c** Western blotting showing PACS-2 levels after PACS-2 knockdown. **d** The relative protein levels of PACS-2. *N* = 300 per group across three replicates. **e** [Ca^2+^]_m_ after PACS-2 knockdown; scale bars, 50 μm. **f** Quantitative analysis of [Ca^2+^]_m_. *N* = 30 per group across three replicates. **g** Quantitative analysis of the immunoblots shown in Fig. 4(i). *N* = 300 per group across three replicates. **h** ROS and GSH levels after PACS-2 knockdown; scale bars, 100 μm. **i** Quantitative analysis of ROS and GSH levels. *N* = 30 per group across three replicates. Graphs show means ± SEM. * *P* < 0.05, ** *P* < 0.01, CD vs. HFD; *t*-test

Pacs-*2* siRNA successfully reduced PACS-2 expression at both the mRNA (Fig. 5a, b) and protein levels (Fig. 5c, d). The level of MAM was significantly decreased by PACS-2 knockdown (Fig. 4e, f). Knocking down PACS-2 led to significantly reduced [Ca^2+^]_m_ (Fig. 5e, f) and decreased cytochrome C and increased P-MAPK 3/1 expression (Fig. 4i and Fig. 5g). This was accompanied by significantly decreased ROS and increased GSH levels (Fig. 5h, i).

## Discussion

Previous studies reported that obesity leads to functional defects in oocytes from both mice and humans [21]. Obesity-induced cellular dysfunction is related to ER stress and mitochondrial dysfunction [22]. However, recent studies demonstrated that the ER and mitochondria interact directly via MAM [16]. Thus, MAM may contribute to these oocyte defects.

The current study revealed that MAM were enriched in oocytes from obese mice. This is consistent with the results of a previous study revealing that MAM are enriched in the hepatocytes of obese mice [19]. Ideally, MAM should be studied using enriched fractions of MAM from oocytes. However, a previous study that used ~7 × 10^7^ cells or 350–400 g liver tissues to isolate enriched fractions of MAM revealed that we would need at least 140,000 oocytes (one oocyte is equivalent to 500 granule cells) to obtain a sufficient amount of enriched fractions for study, which is the equivalent of ~4000 mice per group; this is not possible [23]. Therefore, we decided to assess whether the levels of MAM-related proteins were changed in oocytes from obese mice. When we measured the mRNA and protein levels of the proteins related to MAM in oocytes, the data revealed a significant increase in IP3R1, IP3R2, and PACS-2 levels.

The MAM-related protein IP3R1 is a major ER Ca^2+^ regulatory protein in oocytes, whereas PACS-2 plays a key role in regulating ER homeostasis and the physical interaction between the ER and mitochondria. Therefore, increased levels of the MAM-related proteins IP3R1 or PACS-2 in oocytes from obese mice might lead to increased ER Ca^2+^ release and [Ca^2+^]_m_ intake [24]. Ca^2+^ is a major regulator of mitochondrial physiology [25]. Abnormal increases in [Ca^2+^]_m_, even a tiny change, lead to subsequent mitochondrial dysfunction and even apoptosis [9, 26]. Indeed, the current results showed that [Ca^2+^]_m_ was increased in oocytes from obese mice. The intrinsic apoptotic pathways related to oocytes were activated, and in particular the levels of the two important proteins cytochrome C and activated caspase-3 were increased. Regarding cytoplasmic maturation, oocytes from obese mice exhibited a significant reduction in GSH and P-MAPK 3/1 levels (the latter is related to microtubule organization) and a significant increase in ROS. All three of these features indicated compromised cytoplasmic maturation.

To assess whether the increased [Ca^2+^]_m_, activated apoptosis pathway, and compromised cytoplasmic maturation in oocytes from obese mice were influenced by the MAM-related proteins IP3R1 or PACS-2, RNAi was used to knock them down. Knocking down either IP3R1 or PACS-2 in oocytes reduced the [Ca^2+^]_m_, relieved apoptosis, and simultaneously decreased the compromised cytoplasmic maturation in oocytes from obese mice. The results also showed that knocking down PACS-2, but not IP3R1, reduced the MAM content. This may because PACS-2 is an integral MAM protein that regulates the ER-mitochondrial physical interaction, whereas IP3R1 only regulates ER-mitochondrial Ca^2+^ fluxes. Therefore, knocking down IP3R1 may just affect the function of MAM rather than their morphology. Knockdown of PACS-2 did reduce MAM content and improve oocyte quality.

## Conclusions

In conclusion, it is possible that enriched MAM could increases [Ca^2+^]_m_, which is associated with increased apoptosis and compromised cytoplasmic maturation of oocytes from obese mice. This finding suggests a novel therapeutic target for obesity-induced oocyte defects.

## Additional files

Additional file 1: Table S1. Formulas of D12450B (CD) and D12492 (HFD). (DOCX 27 kb)

Additional file 2: Figure S1. Female CD-1 mice exhibit obesity after being fed a HFD for 12 weeks. (a) Representative image of female CD-1 mice fed a CD or HFD diet for 12 weeks. (b) The mean bodyweight of mice in the HFD group was higher than that of mice in the CD group. *N* = 105 for HFD group and *N* = 112 for CD group across three replicates. (c) The mean bodyweight of mice in both groups changed over time. *N* = 105 for HFD group and *N* = 112 for CD group across three replicates. (d) The proportion of abdominal adipose weight in both groups. *N* = 18 for each group across three replicates. Graphs show means ± SEM. * *P* < 0.05, ** *P* < 0.01, *t*-test. (TIFF 1850 kb)

Additional file 3: Table S2. List of the primers used for qRT-PCR. (DOCX 17 kb)

## Abbreviations

[Ca^2+^]_m_: Mitochondrial Ca^2+^
CD: Control diet
ER: Endoplasmic reticulum
HFD: High fat diet
IP3R1: Inositol 1,4,5-trisphosphate receptor, type 1
IP3R2: Inositol 1,4,5-trisphosphate receptor, type 2
MAM: Mitochondria-associated ER membranes
Mfn2: Mitofusin-2
PACS–2: Phosphofurin acidic cluster sorting protein 2
P-MAPK 3/1: Phosphorylation of extracellular-regulated protein kinases 3/1
Sig-1R: Sigma-1 receptor
