# Supplementary material for: Enriched endoplasmic reticulum-mitochondria interactions result in mitochondrial dysfunction and apoptosis in oocytes from obese mice
Source: J Anim Sci Biotechnol. 2017 Aug 1;8:62. doi: 10.1186/s40104-017-0195-z (PMC5537973; doi:10.1186/s40104-017-0195-z)
Supplement: Supplementary file 1 — Formulas of D12450B (CD) and D12492 (HFD). (DOCX 27 kb) [file 40104_2017_195_MOESM1_ESM.docx]

**Table S1. Formulas of D12450B (CD) and D12492 (HFD)**

| **Ingredient** | **CD** | **HFD** |
| --- | --- | --- |
|  | gm% | |
| Protein | 19.2 | 26.2 |
| Carbohydrate | 67.3 | 26.3 |
| Fat | 4.3 | 34.9 |
|  | gm | |
| Casein | 200 | 200 |
| L-Cystine | 3 | 3 |
| Corn Starch | 315 | 0 |
| Maltodextrin | 35 | 125 |
| Sucrose | 350 | 68.8 |
| Cellulose | 50 | 50 |
| Soybean Oil | 25 | 25 |
| Lard | 20 | 245 |
| Mineral Mix | 10 | 10 |
| DiCalcium Phosphate | 13 | 13 |
| Calcium Carbonate | 5.5 | 5.5 |
| Potassium Citrate | 16.5 | 16.5 |
| Vitamin Mix | 10 | 10 |
| Choline Bitartrate | 2 | 2 |
| **Total** | **1055.05** | **773.85** |

*CD and HFD were with 10% and 60% kcal% fat respectively. They were both used in Obesity Research, and CD acted as a control diet.
