# Supplementary figures and images for: Enriched endoplasmic reticulum-mitochondria interactions result in mitochondrial dysfunction and apoptosis in oocytes from obese mice
Source: J Anim Sci Biotechnol. 2017 Aug 1;8:62. doi: 10.1186/s40104-017-0195-z (PMC5537973; doi:10.1186/s40104-017-0195-z)

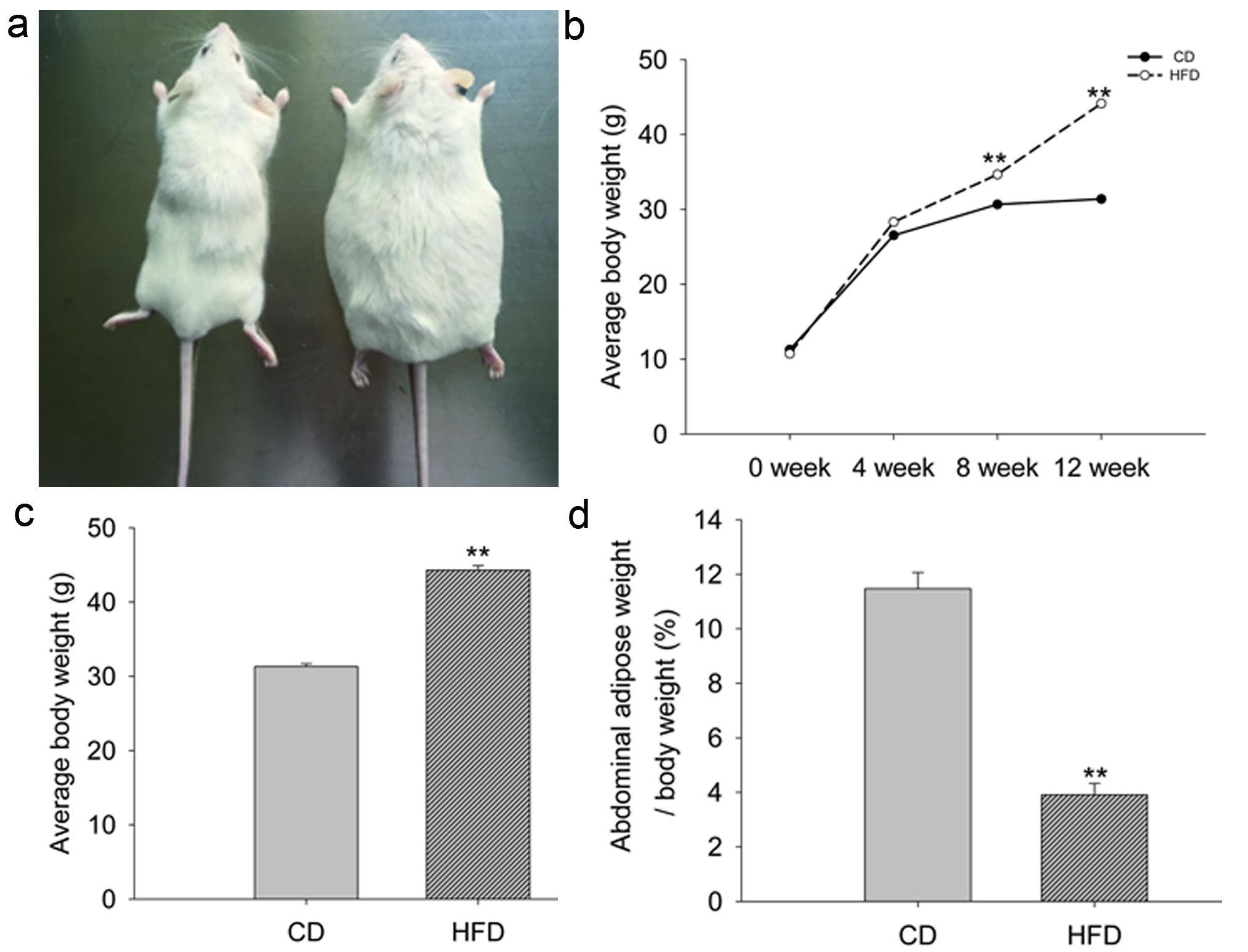

Supplement: Supplementary file 2 — Female CD-1 mice exhibit obesity after being fed a HFD for 12 weeks. (a) Representative image of female CD-1 mice fed a CD or HFD diet for 12 weeks. (b) The mean bodyweight of mice in the HFD group was higher than that of mice in the CD group. N = 105 for HFD group and N = 112 for CD group across three replicates. (c) The mean bodyweight of mice in both groups changed over time. N = 105 for HFD group and N = 112 for CD group across three replicates. (d) The proportion of abdominal adipose weight in both groups. N = 18 for each group across three replicates. Graphs show means ± SEM. * P < 0.05, ** P < 0.01, t-test. (TIFF 1850 kb) [file 40104_2017_195_MOESM2_ESM.tif]
