# Supplementary material for: Enriched endoplasmic reticulum-mitochondria interactions result in mitochondrial dysfunction and apoptosis in oocytes from obese mice
Source: J Anim Sci Biotechnol. 2017 Aug 1;8:62. doi: 10.1186/s40104-017-0195-z (PMC5537973; doi:10.1186/s40104-017-0195-z)
Supplement: Supplementary file 3 — List of the primers used for qRT-PCR. (DOCX 17 kb) [file 40104_2017_195_MOESM3_ESM.docx]

**Table S2. List of primers utilized for qRT-PCR**

| **Genes** | **Forwards (5’……3’)** | **Backwards (5’……3’)** |
| --- | --- | --- |
| *Itpr1* | CCTGTGGGAAGTGGAGGTAG | GCCAAGTAATGCCCTGTAGC |
| *Itpr2* | CCTCTACATTGGGGACATCGT | GGCACACCTTGAACAGGCA |
| *Pacs-2* | GCAAGTGGAAAGGCTGGTAA | AGCAAAGGGCAGGATACAAA |
| *Mfn2* | GCCCTCTCCTTTGGACTGTA | GCTTCTCACTGGCGTATTCC |
| *Sigmar1* | GGCACCACGAAAAGTGAGGT | AGAACAGGGTAGACGGAATAACA |
| *Canx* | CTTCCAGGGGATAAAGGACTTGT | ACATAGGCACCACCACATTCTA |
| *Actin* | CTAAGGCCAACCGTGAAAAG | ACCAGAGGCATACAGGGACA |
